# {Tris[2-(5-bromo-2-oxidobenzyl­idene­amino)eth­yl]amine}manganese(III)

**DOI:** 10.1107/S160053680804172X

**Published:** 2008-12-13

**Authors:** In-Chul Hwang, Kwang Ha

**Affiliations:** a Department of Chemistry, Pohang University of Science and Technology, Pohang 790-784, Republic of Korea; b School of Applied Chemical Engineering, The Research Institute of Catalysis, Chonnam National University, Gwangju 500-757, Republic of Korea

## Abstract

In the title complex, [Mn(C_27_H_24_Br_3_N_4_O_3_)], the Mn^III^ ion is six-coordinated in a distorted octa­hedral environment by three N atoms and three O atoms from the trianion of the hexa­dentate ligand tris­[2-(5-bromo-2-oxidobenzyl­idene­amino)eth­yl]amine. All three N (and O) atoms are *cis* to each other. The three N and the three O atoms are in a *fac* conformation among each other.

## Related literature

For related literature, see: Hwang & Ha (2007 ▶); Mitra *et al.* (2006 ▶).
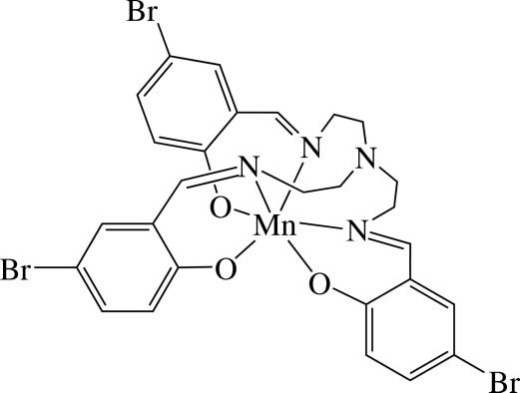


## Experimental

### 

#### Crystal data

[Mn(C_27_H_24_Br_3_N_4_O_3_)]
*M* *_r_* = 747.17
Triclinic, 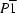

*a* = 9.5892 (15) Å
*b* = 11.7558 (18) Å
*c* = 13.417 (2) Å
α = 80.041 (3)°
β = 78.084 (3)°
γ = 89.069 (3)°
*V* = 1457.3 (4) Å^3^
*Z* = 2
Mo *K*α radiation
μ = 4.60 mm^−1^
*T* = 293 (2) K
0.25 × 0.15 × 0.10 mm

#### Data collection

Bruker SMART 1000 CCD diffractometer
Absorption correction: multi-scan (*SADABS*; Bruker, 2000 ▶) *T*_min_ = 0.422, *T*_max_ = 0.631
9621 measured reflections
5820 independent reflections
3794 reflections with *I* > 2σ(*I*)
*R*_int_ = 0.025

#### Refinement

*R*[*F* ^2^ > 2σ(*F* ^2^)] = 0.067
*wR*(*F* ^2^) = 0.238
*S* = 1.04
5820 reflections
343 parameters
H-atom parameters constrained
Δρ_max_ = 2.66 e Å^−3^
Δρ_min_ = −0.52 e Å^−3^

### 

Data collection: *SMART* (Bruker, 2000 ▶); cell refinement: *SAINT* (Bruker, 2000 ▶); data reduction: *SAINT*; program(s) used to solve structure: *SHELXS97* (Sheldrick, 2008 ▶); program(s) used to refine structure: *SHELXL97* (Sheldrick, 2008 ▶); molecular graphics: *ORTEP-3* (Farrugia, 1997 ▶) and *PLATON* (Spek, 2003 ▶); software used to prepare material for publication: *SHELXL97*.

## Supplementary Material

Crystal structure: contains datablocks global, I. DOI: 10.1107/S160053680804172X/bt2817sup1.cif

Structure factors: contains datablocks I. DOI: 10.1107/S160053680804172X/bt2817Isup2.hkl

Additional supplementary materials:  crystallographic information; 3D view; checkCIF report

## Figures and Tables

**Table:** 

Mn—O2	1.884 (5)
Mn—O1	1.905 (5)
Mn—N3	2.064 (6)
Mn—N2	2.073 (6)
Mn—O3	2.105 (5)
Mn—N1	2.369 (6)

**Table:** 

O2—Mn—N3	169.7 (2)
O1—Mn—N2	171.2 (2)
O3—Mn—N1	169.35 (19)

**Fig. 1.**
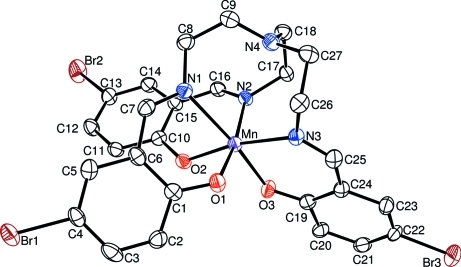
The structure of the title compound with the numbering scheme. Displacement ellipsoids are drawn at the 30% probability level. H atoms have been omitted for clarity.

**Fig. 2.**
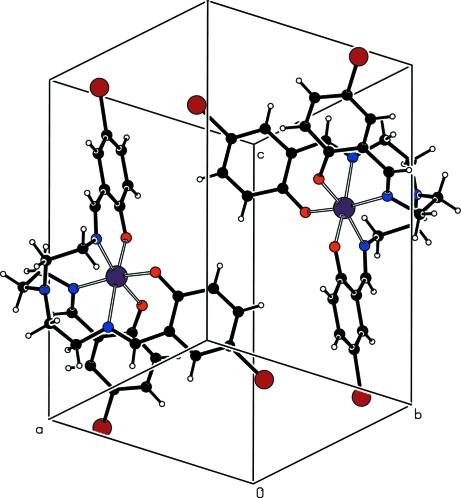
View of the unit-cell contents of the title complex.

## supplementary crystallographic information

### Comment

In the title complex, [Mn(C_27_H_24_Br_3_N_4_O_3_)], the Mn^3+^ ion is six-coordinated in a distorted octahedral environment by three N atoms and three O atoms from the trianion of the hexadentate ligand *N*,*N*',*N*"-tris(5-bromosalicylidene)tris(2-aminoethyl)amine. All three N (and O) atoms are adjacent and lie in the facial position (Fig.1 and Fig.2). The apical O1—Mn—N2, O2—Mn—N3 and O3—Mn—N1 bond angles are 171.2 (2)°, 169.7 (2)° and 169.35 (19)°, respectively (Table 1). The Mn—N bonds are on average 0.204 Å longer than the Mn—O bonds (mean lengths: Mn—N 2.169 Å, Mn—O 1.965 Å).

### Experimental

Mn(CH_3_COO)_3_.2H_2_O (0.50 g, 1.86 mmol) and *N*,*N*',*N*"-tris(5-bromosalicylidene)tris(2-aminoethyl)amine (Brsaltren; 1.30 g, 1.87 mmol) in EtOH (70 ml) were stirred for 3 h at room temparature. The formed precipitate was separated by filtration and washed with acetone, and dried under vacuum, to give a dark green powder (1.16 g). Crystals suitable for X-ray analysis were obtained by slow evaporation from an acetone/EtOH solution. MS (FAB): m/*z* 746, 748, 750, 752 (Mn(Brsaltren-H)^+^); IR (KBr): 3448 cm^-1^ (broad).

### Refinement

H atoms were positioned geometrically and allowed to ride on their respective carrier atoms [C—H = 0.93 (CH) or 0.97 Å (CH_2_) and *U*_iso_(H) = 1.2*U*_eq_(C)]. The CIF check program indicates a high ratio of the maximum and minimum residual density (5.09) in the structure and solvent accessible voids of 142 Å^3^. All these factors indicate a strong likelihood of disordered solvent molecules acetone or EtOH in the structure. However, the solvent molecule could neither be located nor refined. The distances between the highest difference peak (2.66 e Å^-3^) and the nearest peaks (1.96, 1.69 and 1.08 e Å^-3^) are 1.114, 1.540 and 1.137 Å.

### Crystal data

**Table:**

[Mn(C_27_H_24_Br_3_N_4_O_3_)]	*Z* = 2
*M**_r_* = 747.17	*F*(000) = 736
Triclinic, *P*1	*D*_x_ = 1.703 Mg m^−^^3^
Hall symbol: -P 1	Mo *K*α radiation, λ = 0.71073 Å
*a* = 9.5892 (15) Å	Cell parameters from 2848 reflections
*b* = 11.7558 (18) Å	θ = 2.8–24.2°
*c* = 13.417 (2) Å	µ = 4.60 mm^−^^1^
α = 80.041 (3)°	*T* = 293 K
β = 78.084 (3)°	Plate, black
γ = 89.069 (3)°	0.25 × 0.15 × 0.10 mm
*V* = 1457.3 (4) Å^3^	

### Data collection

**Table:**

Bruker SMART 1000 CCD diffractometer	5820 independent reflections
Radiation source: fine-focus sealed tube	3794 reflections with *I* > 2σ(*I*)
graphite	*R*_int_ = 0.025
φ and ω scans	θ_max_ = 26.4°, θ_min_ = 1.6°
Absorption correction: multi-scan (*SADABS*; Bruker, 2000)	*h* = −11→11
*T*_min_ = 0.422, *T*_max_ = 0.631	*k* = −10→14
9621 measured reflections	*l* = −16→16

### Refinement

**Table:**

Refinement on *F*^2^	Primary atom site location: structure-invariant direct methods
Least-squares matrix: full	Secondary atom site location: difference Fourier map
*R*[*F*^2^ > 2σ(*F*^2^)] = 0.067	Hydrogen site location: inferred from neighbouring sites
*wR*(*F*^2^) = 0.238	H-atom parameters constrained
*S* = 1.04	*w* = 1/[σ^2^(*F*_o_^2^) + (0.1477*P*)^2^] where *P* = (*F*_o_^2^ + 2*F*_c_^2^)/3
5820 reflections	(Δ/σ)_max_ < 0.001
343 parameters	Δρ_max_ = 2.66 e Å^−^^3^
0 restraints	Δρ_min_ = −0.52 e Å^−^^3^

### Special details

**Table:**

Geometry. All e.s.d.'s (except the e.s.d. in the dihedral angle between two l.s. planes) are estimated using the full covariance matrix. The cell e.s.d.'s are taken into account individually in the estimation of e.s.d.'s in distances, angles and torsion angles; correlations between e.s.d.'s in cell parameters are only used when they are defined by crystal symmetry. An approximate (isotropic) treatment of cell e.s.d.'s is used for estimating e.s.d.'s involving l.s. planes.
Refinement. Refinement of *F*^2^ against ALL reflections. The weighted *R*-factor *wR* and goodness of fit *S* are based on *F*^2^, conventional *R*-factors *R* are based on *F*, with *F* set to zero for negative *F*^2^. The threshold expression of *F*^2^ > σ(*F*^2^) is used only for calculating *R*-factors(gt) *etc*. and is not relevant to the choice of reflections for refinement. *R*-factors based on *F*^2^ are statistically about twice as large as those based on *F*, and *R*- factors based on ALL data will be even larger.

### Fractional atomic coordinates and isotropic or equivalent isotropic displacement parameters (Å^2^)

**Table:**

	*x*	*y*	*z*	*U*_iso_*/*U*_eq_	
Mn	0.89446 (11)	0.29092 (8)	0.37296 (8)	0.0339 (3)	
Br1	0.43752 (9)	0.63459 (7)	0.07601 (7)	0.0562 (3)	
Br2	0.65915 (12)	−0.10364 (9)	0.06388 (9)	0.0773 (4)	
Br3	0.92383 (12)	0.21717 (12)	0.94128 (7)	0.0817 (4)	
O1	0.8231 (6)	0.4437 (4)	0.3637 (4)	0.0440 (13)	
O2	0.7336 (5)	0.2435 (4)	0.3291 (4)	0.0390 (12)	
O3	0.7913 (6)	0.2169 (4)	0.5240 (4)	0.0438 (12)	
N1	0.9821 (6)	0.3512 (5)	0.1933 (4)	0.0379 (14)	
N2	0.9811 (6)	0.1304 (5)	0.3586 (4)	0.0345 (13)	
N3	1.0442 (6)	0.3553 (5)	0.4404 (5)	0.0371 (14)	
N4	1.2302 (6)	0.2689 (5)	0.2753 (5)	0.0421 (14)	
C1	0.7448 (8)	0.4866 (6)	0.2978 (6)	0.0386 (16)	
C2	0.6213 (9)	0.5503 (7)	0.3293 (6)	0.0471 (19)	
H2	0.5994	0.5618	0.3976	0.056*	
C3	0.5340 (9)	0.5950 (7)	0.2667 (7)	0.055 (2)	
H3	0.4540	0.6354	0.2917	0.066*	
C4	0.5650 (8)	0.5800 (6)	0.1638 (6)	0.0423 (18)	
C5	0.6844 (9)	0.5206 (7)	0.1259 (6)	0.0475 (19)	
H5	0.7041	0.5118	0.0569	0.057*	
C6	0.7768 (8)	0.4732 (6)	0.1912 (6)	0.0416 (17)	
C7	0.8986 (8)	0.4149 (6)	0.1456 (6)	0.0434 (18)	
H7	0.9195	0.4240	0.0738	0.052*	
C8	1.0976 (8)	0.2977 (7)	0.1326 (6)	0.0445 (18)	
H8A	1.1076	0.3320	0.0603	0.053*	
H8B	1.0754	0.2161	0.1395	0.053*	
C9	1.2362 (8)	0.3111 (7)	0.1653 (6)	0.0462 (19)	
H9A	1.3090	0.2695	0.1251	0.055*	
H9B	1.2643	0.3922	0.1495	0.055*	
C10	0.7244 (7)	0.1687 (6)	0.2679 (5)	0.0332 (15)	
C11	0.6176 (8)	0.1814 (7)	0.2102 (6)	0.0440 (18)	
H11	0.5571	0.2439	0.2131	0.053*	
C12	0.6016 (8)	0.1000 (7)	0.1481 (6)	0.0465 (19)	
H12	0.5314	0.1092	0.1090	0.056*	
C13	0.6884 (8)	0.0077 (7)	0.1451 (6)	0.0451 (19)	
C14	0.7932 (8)	−0.0056 (6)	0.1990 (6)	0.0422 (18)	
H14	0.8513	−0.0695	0.1953	0.051*	
C15	0.8169 (7)	0.0738 (6)	0.2602 (5)	0.0350 (15)	
C16	0.9320 (8)	0.0567 (6)	0.3142 (6)	0.0386 (16)	
H16	0.9751	−0.0149	0.3172	0.046*	
C17	1.1154 (7)	0.0991 (6)	0.3940 (6)	0.0381 (16)	
H17A	1.1218	0.0157	0.4099	0.046*	
H17B	1.1168	0.1301	0.4562	0.046*	
C18	1.2411 (8)	0.1481 (6)	0.3092 (6)	0.0430 (18)	
H18A	1.3284	0.1337	0.3348	0.052*	
H18B	1.2466	0.1085	0.2509	0.052*	
C19	0.8246 (8)	0.2186 (6)	0.6102 (6)	0.0398 (17)	
C20	0.7371 (9)	0.1565 (7)	0.7023 (6)	0.0442 (18)	
H20	0.6570	0.1161	0.6971	0.053*	
C21	0.7678 (9)	0.1546 (7)	0.8001 (6)	0.050 (2)	
H21	0.7097	0.1128	0.8588	0.060*	
C22	0.8866 (9)	0.2160 (7)	0.8083 (6)	0.049 (2)	
C23	0.9756 (9)	0.2779 (7)	0.7228 (6)	0.0463 (19)	
H23	1.0542	0.3187	0.7299	0.056*	
C24	0.9449 (8)	0.2781 (6)	0.6232 (6)	0.0382 (16)	
C25	1.0426 (9)	0.3453 (6)	0.5379 (6)	0.0421 (18)	
H25	1.1132	0.3866	0.5554	0.051*	
C26	1.1647 (8)	0.4255 (6)	0.3728 (6)	0.0451 (18)	
H26A	1.2006	0.4773	0.4111	0.054*	
H26B	1.1332	0.4717	0.3146	0.054*	
C27	1.2823 (8)	0.3454 (7)	0.3340 (6)	0.0460 (19)	
H27A	1.3628	0.3909	0.2906	0.055*	
H27B	1.3144	0.3002	0.3923	0.055*	

### Atomic displacement parameters (Å^2^)

**Table:**

	*U*^11^	*U*^22^	*U*^33^	*U*^12^	*U*^13^	*U*^23^
Mn	0.0380 (6)	0.0287 (6)	0.0393 (6)	0.0037 (4)	−0.0134 (5)	−0.0116 (5)
Br1	0.0540 (5)	0.0463 (5)	0.0767 (6)	0.0125 (4)	−0.0318 (5)	−0.0126 (4)
Br2	0.0940 (8)	0.0649 (7)	0.0999 (9)	0.0106 (6)	−0.0553 (7)	−0.0468 (6)
Br3	0.0812 (8)	0.1293 (11)	0.0422 (6)	0.0090 (7)	−0.0231 (5)	−0.0234 (6)
O1	0.053 (3)	0.035 (3)	0.054 (3)	0.010 (2)	−0.025 (3)	−0.020 (2)
O2	0.037 (3)	0.038 (3)	0.048 (3)	0.008 (2)	−0.013 (2)	−0.017 (2)
O3	0.048 (3)	0.050 (3)	0.037 (3)	−0.005 (2)	−0.015 (2)	−0.012 (2)
N1	0.039 (3)	0.038 (3)	0.038 (3)	0.005 (3)	−0.008 (3)	−0.008 (3)
N2	0.031 (3)	0.034 (3)	0.041 (3)	0.002 (2)	−0.010 (3)	−0.010 (3)
N3	0.045 (4)	0.028 (3)	0.043 (4)	0.003 (3)	−0.014 (3)	−0.012 (3)
N4	0.039 (3)	0.039 (4)	0.048 (4)	0.000 (3)	−0.010 (3)	−0.006 (3)
C1	0.040 (4)	0.028 (4)	0.050 (4)	0.000 (3)	−0.012 (3)	−0.011 (3)
C2	0.057 (5)	0.044 (4)	0.044 (4)	0.008 (4)	−0.013 (4)	−0.017 (4)
C3	0.043 (5)	0.042 (5)	0.088 (7)	0.014 (4)	−0.022 (4)	−0.026 (4)
C4	0.044 (4)	0.029 (4)	0.059 (5)	0.008 (3)	−0.023 (4)	−0.008 (3)
C5	0.057 (5)	0.043 (5)	0.046 (5)	0.001 (4)	−0.020 (4)	−0.005 (4)
C6	0.042 (4)	0.032 (4)	0.052 (5)	0.004 (3)	−0.015 (4)	−0.004 (3)
C7	0.048 (5)	0.045 (4)	0.039 (4)	−0.001 (4)	−0.009 (4)	−0.014 (3)
C8	0.049 (5)	0.044 (4)	0.041 (4)	0.008 (4)	−0.007 (3)	−0.010 (3)
C9	0.039 (4)	0.045 (5)	0.052 (5)	0.004 (3)	−0.006 (4)	−0.007 (4)
C10	0.033 (4)	0.030 (4)	0.036 (4)	−0.008 (3)	−0.004 (3)	−0.006 (3)
C11	0.036 (4)	0.050 (5)	0.051 (5)	0.008 (3)	−0.011 (3)	−0.021 (4)
C12	0.039 (4)	0.048 (5)	0.059 (5)	0.001 (3)	−0.020 (4)	−0.017 (4)
C13	0.050 (5)	0.040 (4)	0.054 (5)	0.000 (4)	−0.021 (4)	−0.023 (4)
C14	0.043 (4)	0.037 (4)	0.050 (5)	0.004 (3)	−0.011 (4)	−0.016 (3)
C15	0.033 (4)	0.031 (4)	0.044 (4)	0.003 (3)	−0.010 (3)	−0.011 (3)
C16	0.040 (4)	0.031 (4)	0.048 (4)	0.008 (3)	−0.012 (3)	−0.015 (3)
C17	0.036 (4)	0.036 (4)	0.046 (4)	0.005 (3)	−0.016 (3)	−0.010 (3)
C18	0.040 (4)	0.041 (4)	0.050 (5)	0.005 (3)	−0.010 (3)	−0.014 (4)
C19	0.044 (4)	0.032 (4)	0.045 (4)	0.004 (3)	−0.010 (3)	−0.013 (3)
C20	0.050 (5)	0.042 (4)	0.044 (4)	0.006 (4)	−0.009 (4)	−0.017 (3)
C21	0.051 (5)	0.054 (5)	0.043 (5)	0.010 (4)	−0.003 (4)	−0.012 (4)
C22	0.058 (5)	0.061 (5)	0.035 (4)	0.016 (4)	−0.017 (4)	−0.024 (4)
C23	0.054 (5)	0.049 (5)	0.042 (4)	0.006 (4)	−0.020 (4)	−0.015 (4)
C24	0.046 (4)	0.030 (4)	0.042 (4)	0.003 (3)	−0.014 (3)	−0.012 (3)
C25	0.056 (5)	0.025 (4)	0.051 (5)	0.005 (3)	−0.019 (4)	−0.013 (3)
C26	0.053 (5)	0.035 (4)	0.047 (5)	−0.008 (4)	−0.009 (4)	−0.008 (3)
C27	0.037 (4)	0.041 (4)	0.059 (5)	−0.007 (3)	−0.005 (4)	−0.011 (4)

### Geometric parameters (Å, °)

**Table:**

Mn—O2	1.884 (5)	C9—H9A	0.9700
Mn—O1	1.905 (5)	C9—H9B	0.9700
Mn—N3	2.064 (6)	C10—C11	1.397 (10)
Mn—N2	2.073 (6)	C10—C15	1.418 (9)
Mn—O3	2.105 (5)	C11—C12	1.403 (10)
Mn—N1	2.369 (6)	C11—H11	0.9300
Br1—C4	1.898 (7)	C12—C13	1.357 (10)
Br2—C13	1.901 (7)	C12—H12	0.9300
Br3—C22	1.892 (7)	C13—C14	1.346 (10)
O1—C1	1.306 (8)	C14—C15	1.396 (9)
O2—C10	1.319 (8)	C14—H14	0.9300
O3—C19	1.265 (8)	C15—C16	1.433 (9)
N1—C7	1.280 (9)	C16—H16	0.9300
N1—C8	1.438 (9)	C17—C18	1.519 (10)
N2—C16	1.278 (9)	C17—H17A	0.9700
N2—C17	1.482 (8)	C17—H17B	0.9700
N3—C25	1.291 (9)	C18—H18A	0.9700
N3—C26	1.476 (10)	C18—H18B	0.9700
N4—C18	1.424 (9)	C19—C24	1.416 (10)
N4—C27	1.449 (9)	C19—C20	1.429 (11)
N4—C9	1.464 (10)	C20—C21	1.400 (10)
C1—C2	1.420 (10)	C20—H20	0.9300
C1—C6	1.435 (10)	C21—C22	1.392 (12)
C2—C3	1.343 (10)	C21—H21	0.9300
C2—H2	0.9300	C22—C23	1.379 (12)
C3—C4	1.392 (12)	C23—C24	1.426 (10)
C3—H3	0.9300	C23—H23	0.9300
C4—C5	1.382 (11)	C24—C25	1.442 (11)
C5—C6	1.414 (10)	C25—H25	0.9300
C5—H5	0.9300	C26—C27	1.523 (10)
C6—C7	1.426 (10)	C26—H26A	0.9700
C7—H7	0.9300	C26—H26B	0.9700
C8—C9	1.501 (10)	C27—H27A	0.9700
C8—H8A	0.9700	C27—H27B	0.9700
C8—H8B	0.9700		
			
O2—Mn—O1	89.3 (2)	C11—C10—C15	118.6 (6)
O2—Mn—N3	169.7 (2)	C10—C11—C12	120.0 (7)
O1—Mn—N3	84.2 (2)	C10—C11—H11	120.0
O2—Mn—N2	88.6 (2)	C12—C11—H11	120.0
O1—Mn—N2	171.2 (2)	C13—C12—C11	120.1 (7)
N3—Mn—N2	98.9 (2)	C13—C12—H12	120.0
O2—Mn—O3	86.9 (2)	C11—C12—H12	120.0
O1—Mn—O3	101.1 (2)	C14—C13—C12	121.0 (7)
N3—Mn—O3	86.5 (2)	C14—C13—Br2	120.2 (6)
N2—Mn—O3	87.3 (2)	C12—C13—Br2	118.9 (6)
O2—Mn—N1	83.2 (2)	C13—C14—C15	121.8 (7)
O1—Mn—N1	82.7 (2)	C13—C14—H14	119.1
N3—Mn—N1	103.9 (2)	C15—C14—H14	119.1
N2—Mn—N1	88.5 (2)	C14—C15—C10	118.5 (6)
O3—Mn—N1	169.35 (19)	C14—C15—C16	119.5 (6)
C1—O1—Mn	123.1 (4)	C10—C15—C16	122.0 (6)
C10—O2—Mn	129.3 (4)	N2—C16—C15	126.4 (6)
C19—O3—Mn	130.7 (5)	N2—C16—H16	116.8
C7—N1—C8	117.9 (6)	C15—C16—H16	116.8
C7—N1—Mn	115.4 (5)	N2—C17—C18	109.2 (6)
C8—N1—Mn	124.4 (5)	N2—C17—H17A	109.8
C16—N2—C17	115.8 (6)	C18—C17—H17A	109.8
C16—N2—Mn	124.4 (5)	N2—C17—H17B	109.8
C17—N2—Mn	119.6 (4)	C18—C17—H17B	109.8
C25—N3—C26	114.3 (6)	H17A—C17—H17B	108.3
C25—N3—Mn	127.1 (5)	N4—C18—C17	112.4 (6)
C26—N3—Mn	118.5 (5)	N4—C18—H18A	109.1
C18—N4—C27	117.0 (6)	C17—C18—H18A	109.1
C18—N4—C9	118.5 (6)	N4—C18—H18B	109.1
C27—N4—C9	117.8 (6)	C17—C18—H18B	109.1
O1—C1—C2	120.7 (7)	H18A—C18—H18B	107.9
O1—C1—C6	123.1 (6)	O3—C19—C24	124.5 (7)
C2—C1—C6	116.2 (7)	O3—C19—C20	119.2 (7)
C3—C2—C1	124.2 (8)	C24—C19—C20	116.3 (7)
C3—C2—H2	117.9	C21—C20—C19	122.2 (7)
C1—C2—H2	117.9	C21—C20—H20	118.9
C2—C3—C4	119.1 (7)	C19—C20—H20	118.9
C2—C3—H3	120.4	C22—C21—C20	119.0 (8)
C4—C3—H3	120.4	C22—C21—H21	120.5
C5—C4—C3	120.7 (7)	C20—C21—H21	120.5
C5—C4—Br1	119.4 (6)	C23—C22—C21	121.9 (7)
C3—C4—Br1	119.8 (6)	C23—C22—Br3	119.3 (6)
C4—C5—C6	120.6 (8)	C21—C22—Br3	118.8 (7)
C4—C5—H5	119.7	C22—C23—C24	118.8 (7)
C6—C5—H5	119.7	C22—C23—H23	120.6
C5—C6—C7	117.0 (7)	C24—C23—H23	120.6
C5—C6—C1	119.2 (7)	C19—C24—C23	121.8 (7)
C7—C6—C1	123.8 (7)	C19—C24—C25	122.9 (6)
N1—C7—C6	126.7 (7)	C23—C24—C25	115.3 (7)
N1—C7—H7	116.7	N3—C25—C24	128.0 (7)
C6—C7—H7	116.7	N3—C25—H25	116.0
N1—C8—C9	112.1 (6)	C24—C25—H25	116.0
N1—C8—H8A	109.2	N3—C26—C27	109.1 (6)
C9—C8—H8A	109.2	N3—C26—H26A	109.9
N1—C8—H8B	109.2	C27—C26—H26A	109.9
C9—C8—H8B	109.2	N3—C26—H26B	109.9
H8A—C8—H8B	107.9	C27—C26—H26B	109.9
N4—C9—C8	113.7 (7)	H26A—C26—H26B	108.3
N4—C9—H9A	108.8	N4—C27—C26	110.2 (6)
C8—C9—H9A	108.8	N4—C27—H27A	109.6
N4—C9—H9B	108.8	C26—C27—H27A	109.6
C8—C9—H9B	108.8	N4—C27—H27B	109.6
H9A—C9—H9B	107.7	C26—C27—H27B	109.6
O2—C10—C11	119.0 (6)	H27A—C27—H27B	108.1
O2—C10—C15	122.4 (6)		
			
O2—Mn—O1—C1	−30.1 (6)	C8—N1—C7—C6	178.5 (7)
N3—Mn—O1—C1	158.0 (6)	Mn—N1—C7—C6	15.1 (10)
O3—Mn—O1—C1	−116.8 (6)	C5—C6—C7—N1	−168.3 (7)
N1—Mn—O1—C1	53.1 (6)	C1—C6—C7—N1	12.2 (12)
O1—Mn—O2—C10	142.7 (6)	C7—N1—C8—C9	133.5 (7)
N3—Mn—O2—C10	−166.1 (11)	Mn—N1—C8—C9	−64.7 (8)
N2—Mn—O2—C10	−28.8 (6)	C18—N4—C9—C8	80.4 (8)
O3—Mn—O2—C10	−116.1 (6)	C27—N4—C9—C8	−129.2 (7)
N1—Mn—O2—C10	59.9 (6)	N1—C8—C9—N4	55.0 (9)
O2—Mn—O3—C19	−173.2 (6)	Mn—O2—C10—C11	−152.4 (6)
O1—Mn—O3—C19	−84.5 (6)	Mn—O2—C10—C15	29.2 (9)
N3—Mn—O3—C19	−1.1 (6)	O2—C10—C11—C12	−177.1 (7)
N2—Mn—O3—C19	98.0 (6)	C15—C10—C11—C12	1.3 (11)
N1—Mn—O3—C19	165.3 (9)	C10—C11—C12—C13	1.0 (12)
O2—Mn—N1—C7	51.5 (5)	C11—C12—C13—C14	−1.8 (13)
O1—Mn—N1—C7	−38.7 (5)	C11—C12—C13—Br2	177.8 (6)
N3—Mn—N1—C7	−120.9 (5)	C12—C13—C14—C15	0.3 (13)
N2—Mn—N1—C7	140.3 (5)	Br2—C13—C14—C15	−179.4 (6)
O3—Mn—N1—C7	73.1 (12)	C13—C14—C15—C10	2.1 (12)
O2—Mn—N1—C8	−110.7 (6)	C13—C14—C15—C16	−178.6 (8)
O1—Mn—N1—C8	159.1 (6)	O2—C10—C15—C14	175.6 (7)
N3—Mn—N1—C8	76.9 (6)	C11—C10—C15—C14	−2.8 (10)
N2—Mn—N1—C8	−21.9 (6)	O2—C10—C15—C16	−3.6 (11)
O3—Mn—N1—C8	−89.1 (12)	C11—C10—C15—C16	177.9 (7)
O2—Mn—N2—C16	11.6 (6)	C17—N2—C16—C15	−170.2 (7)
N3—Mn—N2—C16	−175.5 (6)	Mn—N2—C16—C15	5.0 (11)
O3—Mn—N2—C16	98.5 (6)	C14—C15—C16—N2	167.7 (7)
N1—Mn—N2—C16	−71.7 (6)	C10—C15—C16—N2	−13.1 (12)
O2—Mn—N2—C17	−173.4 (5)	C16—N2—C17—C18	92.1 (7)
N3—Mn—N2—C17	−0.5 (5)	Mn—N2—C17—C18	−83.4 (6)
O3—Mn—N2—C17	−86.5 (5)	C27—N4—C18—C17	85.2 (8)
N1—Mn—N2—C17	103.3 (5)	C9—N4—C18—C17	−124.2 (7)
O2—Mn—N3—C25	46.9 (15)	N2—C17—C18—N4	53.6 (8)
O1—Mn—N3—C25	98.5 (6)	Mn—O3—C19—C24	2.1 (11)
N2—Mn—N3—C25	−89.8 (6)	Mn—O3—C19—C20	−177.6 (5)
O3—Mn—N3—C25	−3.1 (6)	O3—C19—C20—C21	179.9 (7)
N1—Mn—N3—C25	179.5 (6)	C24—C19—C20—C21	0.1 (10)
O2—Mn—N3—C26	−130.0 (12)	C19—C20—C21—C22	0.7 (11)
O1—Mn—N3—C26	−78.4 (5)	C20—C21—C22—C23	−0.6 (12)
N2—Mn—N3—C26	93.3 (5)	C20—C21—C22—Br3	177.9 (6)
O3—Mn—N3—C26	−180.0 (5)	C21—C22—C23—C24	−0.3 (12)
N1—Mn—N3—C26	2.6 (5)	Br3—C22—C23—C24	−178.8 (5)
Mn—O1—C1—C2	135.6 (6)	O3—C19—C24—C23	179.2 (7)
Mn—O1—C1—C6	−44.6 (9)	C20—C19—C24—C23	−1.1 (10)
O1—C1—C2—C3	−178.6 (7)	O3—C19—C24—C25	0.8 (11)
C6—C1—C2—C3	1.5 (12)	C20—C19—C24—C25	−179.4 (6)
C1—C2—C3—C4	−0.6 (13)	C22—C23—C24—C19	1.2 (11)
C2—C3—C4—C5	−0.5 (13)	C22—C23—C24—C25	179.7 (7)
C2—C3—C4—Br1	176.4 (6)	C26—N3—C25—C24	−176.3 (7)
C3—C4—C5—C6	0.5 (12)	Mn—N3—C25—C24	6.7 (11)
Br1—C4—C5—C6	−176.3 (6)	C19—C24—C25—N3	−5.6 (12)
C4—C5—C6—C7	−179.1 (7)	C23—C24—C25—N3	176.0 (7)
C4—C5—C6—C1	0.5 (12)	C25—N3—C26—C27	97.6 (7)
O1—C1—C6—C5	178.8 (7)	Mn—N3—C26—C27	−85.1 (7)
C2—C1—C6—C5	−1.4 (11)	C18—N4—C27—C26	−123.4 (7)
O1—C1—C6—C7	−1.7 (12)	C9—N4—C27—C26	85.7 (8)
C2—C1—C6—C7	178.1 (7)	N3—C26—C27—N4	60.4 (8)
